# Influence of Cooking (Microwaving and Broiling) on Cylindrospermopsin Concentration in Muscle of Nile Tilapia (*Oreochromis niloticus*) and Characterization of Decomposition Products

**DOI:** 10.3390/toxins9060177

**Published:** 2017-05-26

**Authors:** Ana I. Prieto, Remedios Guzmán-Guillén, Rocío Valderrama-Fernández, Ángeles Jos, Ana M. Cameán

**Affiliations:** 1 Area of Toxicology, Faculty of Pharmacy, University of Sevilla, C/Profesor García González 2, 41012 Sevilla, Spain; anaprieto@us.es (A.I.P.); angelesjos@us.es (A.J.); camean@us.es (A.M.C.); 2 Mass spectrometry Facility, Centro de Investigación Tecnológica e Investigación (CITIUS), University of Sevilla, 41012 Sevilla, Spain; rociovalderrama@us.es

**Keywords:** cylindrospermopsin, Nile tilapia, microwaving, broiling, decomposition products

## Abstract

Cylindrospermopsin (CYN) has become increasingly important as a freshwater algal toxin, showing cytotoxic effects. This toxin is able to bioaccumulate in freshwater food webs, representing a serious human health problem. Normally, fish is cooked before consumption, and CYN concentration can be altered. For the first time, the effects of microwaving and broiling for 1 and 2 min on CYN concentration and its decomposition products in fish muscle (*Oreochromis niloticus*) contaminated in the laboratory were investigated, using UPLC-MS/MS and Orbitrap. The results show that cooking the fish reduced unconjugated CYN levels by 11, 10 and 15% after microwaving for 1 and 2 min, and broiling for 2 min, respectively, compared to control fish. Different CYN decomposition products with *m*/*z* 416.1234 (7-epi-CYN) and *m*/*z* 336.16663 (diasteroisomers C-3A, C-3C, C-3D, C-3E, C-3F) are generated in fish samples submitted to cooking. Based on the relative abundance of the decomposition products, the possible degradation pathways taking place by microwaving may be through the formation of 7-epi-CYN and *m*/*z* 336.16663 compounds, whereas in the case of broiling the last route is the only one observed in this study. The influence of cooking and the toxicity characterization of the degradation products generated in CYN-contaminated fish are of importance for more realistic risk evaluation related to their consumption.

## 1. Introduction

Cylindrospermopsin (CYN) has become one of the most important cyanotoxins in fresh waters worldwide due to its frequency of occurrence, different toxicity targets, and impacts on health [1]. Nowadays, there are at least five natural alkaloid toxins recognized as CYNs (CYN, 7-epiCYN, 7-deoxy-CYN, 7-deoxy-desulfo-CYN and 7-deoxy-desulfo-12-acetyl-CYN) [2] produced by *Anabaena* sp., *Aphanizomenon* sp., *Chrysosporum* sp., *Cylindrospermopsis* sp., *Lyngbya* sp., *Raphidiopsis* sp. and *Umezakia* sp. [3]. CYN is an alkaloid, a tricyclic guanidine moiety with a hydroxymethyluracil group, weighting 415 daltons [4]. Its zwitterionic structure makes the toxin highly water-soluble [5], and it has been reported to be stable to temperature, light and pH changes [6].

CYN is cytotoxic, hepatoxic, neurotoxic, genotoxic and possibly induces carcinogenic effects [7,8]. The identification of CYN as a human health hazard was first noticed in an incident which occurred in Palm Island, Australia (1979); the illness included headache, fever, anorexia, vomiting, abdominal pain, constipation and hepatomegaly [1]. The occurrence, bioaccumulation and trophic transference of CYN have been widely shown in food chains in freshwater organisms, including trophic transference to vertebrate (e.g., fish) and invertebrate species (e.g., shellfish) consumed by humans [9], assuming potential risks for human health [10,11,12]. Nevertheless, these data are usually based on raw fish, and fish is not normally consumed raw without processing or cooking. The content of CYN in food may vary due to these procedures [13], which can indeed generate decomposition products of CYN [14], modifying the final consumer exposure to the toxin in a quantitative and qualitative way. Thus, it is necessary to achieve a more accurate knowledge on the actual intake of CYN and its degradation products for a correct consumer safety assessment.

Regarding cyanotoxins, there are only documented studies on the effects of cooking on microcystins (MCs), nodularin (NOD), paralytic shellfish toxins (PSP-toxins) and CYN in clams, mussels, scallops, prawns and fish [13,14,15,16,17,18,19,20,21]. Different patterns of variation for MC levels have been shown in several studies. Guzmán-Guillén et al. [20] showed a reduction on MC-LR (59.3%), MC-YR (56.4%) and MC-RR (45.0%) in fish after boiling, while other authors found a significant increase of MCs in bighead carp and clams [19,21], and no significant changes were found in MC content in mussels after boiling [16]. Moreover, it has been shown that the microwave treatment (for 5 min) significantly reduced the MC content in different aquatic organisms (fish, mussels and clams) [16,20,21]. Bruno et al. [17] observed a partial degradation of MCs after stir-frying (36.3%) and braising (81.6%) fish and a significant increase in baked fish (28%).

Nowadays, only two studies regarding the effects of cooking on CYN content have been performed, in edible mussels and fish (tilapia, *Oreochromis niloticus*), showing different results. Freitas et al. [13] did not show significant alterations in toxin concentration after boiling, microwaving or steaming processes in edible mussels. On the contrary, cooking the fish reduced unconjugated CYN levels by boiling (9 or 18%) and steaming (8 or 26%) for 1 or 2 min, respectively, compared to control fish [14]. Moreover, Adamski et al. [22,23] studied CYN stability and observed its degradation products in cyanobacterial cultures exposed to the influence of temperature and irradiation. In this sense, seven decomposition products were detected in boiled and steamed fish samples (*O. niloticus*) suggesting the plausible CYN decomposition pathways under certain cooking conditions [14]. Therefore, the exact effects of several cooking and eating practices on cyanotoxin levels and exposure remain uncertain, and in fact some international agencies, such as the European Food Safety Authority (EFSA) highlight the need to carry them out [24].

Taking into account all this background, the aim of this study was to investigate: (1) the effects of microwaving and broiling for two different periods of time (1 and 2 min) on the CYN concentration in fish muscle (*O. niloticus*) injected with 50 ng CYN/g dry weight (d.w.), using UPLC-MS/MS and (2) CYN decomposition products in fish fillets injected with 40 µg CYN/g d.w., by UHPLC-MS/MS Orbitrap. As far as we are concerned, this is the first work investigating these two cooking practices with this double purpose.

## 2. Results and Discussion

For an accurate estimation of contaminant intake, it is important to take into account the fact that fish is normally cooked before consumption, and the effects of these treatments which could alter the availability of different contaminants in food should be taken into account [25]. Analysis of the available data by the EFSA indicates that additional efforts should be made to elucidate the levels of human exposure to cyanotoxins under different scenarios. The studies focusing on the influence of different methods of cooking on CYN concentration in edible aquatic animals are necessary to achieve a more accurate knowledge of the actual intake. This would help to perform a correct consumer safety assessment, data very scarce at present [13,14].

**Figure 1:**
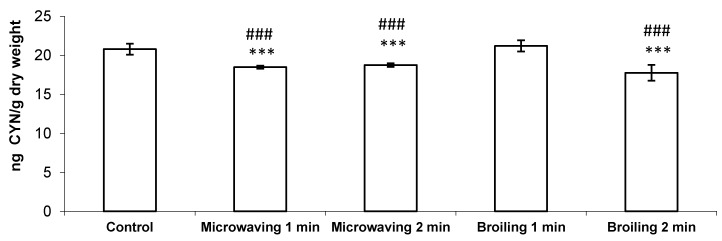
Concentration of CYN (ng CYN/g dry weight) detected in fish muscle (*Oreochromis niloticus*) injected with 500 µL of a CYN standard solution containing 100 µg CYN/L (equivalent to 50 ng CYN/g d.w.) and submitted to either no treatment or to different cooking treatments for 1 and 2 min (microwaving and broiling). Values are expressed as the mean ± SD (*n* = 5). The significant levels observed are *** *p* < 0.001 in comparison to control group (injected and uncooked fish) and ### *p* < 0.001 in comparison to broiling for 1 min. CYN: cylindrospermopsin; d.w.: dry weight; SD: standard deviation.

This work shows for the first time the effects of microwaving and broiling (for 1 or 2 min) on the concentration of unconjugated CYN in fish and the characterization of their decomposition products. The highest effectiveness in reducing CYN levels in cooked fish was obtained by broiling the fish muscle for 2 min (15% reduction) followed by microwaving for 1 min (11% reduction) or 2 min (10% reduction), and there were no significant differences between both cooking times in microwaving (Figure 1). However, broiling treatment for 1 min did not produce any significant reduction in unconjugated CYN in fish muscles, showing a clear influence of the cooking time in this cooking treatment. In this sense, it seems that boiling, steaming and microwaving (0.5–15 min) were not able to alter the availability of CYN in mussels fed CYN-producing *Cylindrospermopsis raciborskii* [13]. By contrast, and in agreement with our results, CYN concentration decreased in fish muscle (Nile tilapia) injected with the toxin and submitted to steaming and boiling for 1 and 2 min compared to the control group [14]. These discrepancies could be explained because the influence of cooking on the CYN levels may depend more on the particular food piece than on the specific cooking practice, as previously suggested by Domingo [25].

Microwave treatment might induce cell fracture [16], inducing hazards mainly because of the heating produced in the tissues [26]. Microwaves allow the temperature reached in the fish to evaporate almost all the water, leading to a high dehydration, and thus weight loss, and consequent matrix modification by protein degradation, as suggested by Freitas et al. [21]. This process might have promoted CYN degradation in the present work, although in edible mussels no effects on CYN concentrations were reported [13]. Previous studies that assessed the influence of microwaves on MC concentration have shown different patterns. The microwave treatment (from 1 to 5 min) significantly reduced MC contents in mussels (*Mytilus galloprovincialis*) [16], while in tilapia fish (*O. niloticus*) 5 min of cooking by microwave was necessary to reduce MC-LR and MC-YR [20]. Later, Freitas et al. [21] found that microwaving for 0.5 and 1 min resulted in higher MC-LR release from the clams (*Corbicula fluminea*), whereas for 3 and 5 min, a significant reduction in toxin concentration was obtained compared to controls. In the present study, microwaving managed to reduce CYN concentrations in fish in shorter periods of time (1 or 2 min), and with the advantage of not obtaining highly dehydrated samples or with physical damage, which would render unappealing organoleptic properties for human consumption, as Freitas et al. [21] described for clams after microwaving for 5 min. These differences could be due to both the different matrices used and the chemical differences between MCs and CYN. Recently, Adamski et al. [23] showed that UV irradiation induced 98 and 100% decomposition of CYN at pH 10 and 12, respectively, also contributing to the formation of some decomposition products. This may suggest that other types of radiation, such as microwaves, could also decrease CYN concentration, similar to the decrease in CYN concentration and presence of decomposition products found in the present work.

Previous studies have shown that CYN is stable under temperature changes (4–50 °C) for up to 5 weeks in the dark; after 4 weeks at 50 °C, 83% of initial (1 mg/L) toxin concentration remained [6]. In the present experiment, temperatures were higher than 100 °C, which could explain the decrease in CYN concentration in the cooking processes. However, the cooking time was a key factor in these results because in the case of broiling, cooking for 1 min did not show differences in CYN content with respect to the control. Similarly, Guzmán-Guillén et al. [14] showed that differences are dependent on the cooking method and the period assayed, with the cooking treatments for 2 min being the most suitable for a significant reduction of CYN levels in the fish muscles. Concerning MCs, Bruno et al. [17] tested different cooking methods to verify MC degradation in muscle of three fish species, showing that braising (similar broiling) resulted in an 82.6% decrease in MC levels, explaining this reduction due to spice presence, temperature or cooking time.

The characterization of the decomposition compounds was only possible when high concentrations of CYN (40 µg CYN/g d.w) were used. The mass spectra of samples revealed: a maximum of 11 decomposition products in samples submitted to microwaving, 7 decomposition products after broiling, with 5 of them (C-1, C-2A, C-2B, 7-epi-CYN, and C-4) being present in all CYN-injected samples (uncooked, microwaving and broiling samples) ([14], Table 1 and Table 2).

Specifically, based on the fragmentation spectra by parallel reaction monitoring (PRM) ([14], Table 1 and Table 2), the obtained decomposition products C-1, C-2B, 7-epi-CYN, C-3A and C-4 coincided with the those previously detected in *C. raciborskii* cultures under high temperature (compounds with *m*/*z* 292.09617, 416.12345 and 336.16663) [22] and under UV-B irradiation in alkaline conditions (compounds with *m*/*z* 290.08052, 292.09617, 416.12345 and 414.10780) [23]. The fragmentation pathways for these products have been proposed by Adamski et al. [22,23]: C1 could derive from the cleavage of the uracil ring, being C-2B a reduced form of it; 7-epi-CYN is a known epimer of CYN; in C-3A the sulfate group of CYN is substituted by a hydroxyl group; and C-4 may come from the hydroxylation with dehydration of CYN.

**Figure 2:**
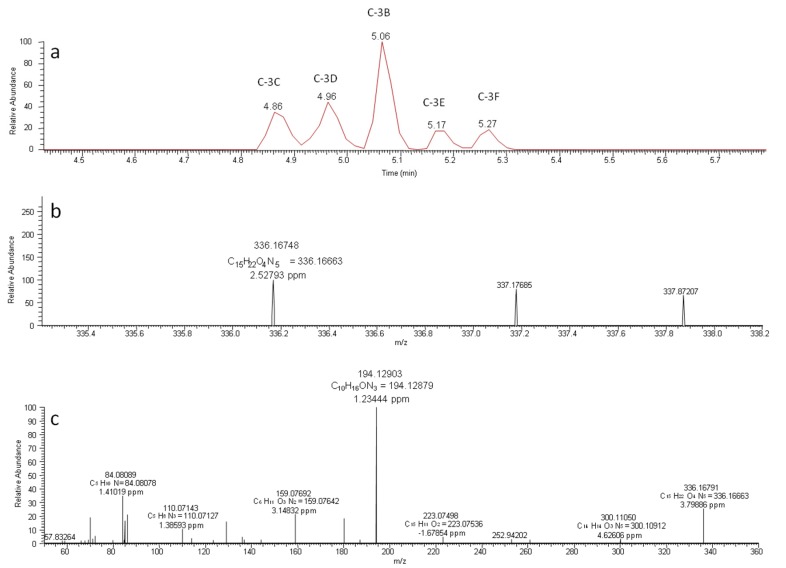
Chromatographic profile (**a**) and mass spectra recorded without fragmentation in FS mode; (**b**) and with fragmentation in PRM mode; (**c**) at 35 eV, of the ion with *m*/*z* 336.16663 with the chemical formula C_15_H_21_N_5_O_4_ in muscle of tilapia fish (*O. niloticus*) cooked by microwave, eluting from 4.86 to 5.27 min.

In this work, as a novelty, a total of six diastereoisomers (C-3A, C-3B, C-3C, C-3D, C-3E and C-3F) (Figure 2) of the decomposition product with *m*/*z* 336.16663 were found in samples submitted to microwaving. These results agree in part with those previously found by Adamski et al. [22] in *C. racibosrkii* cultures under the influence of temperature and pH, who reported four decomposition products for the same molecular ion at *m*/*z* 336.16663.

**Figure 3:**
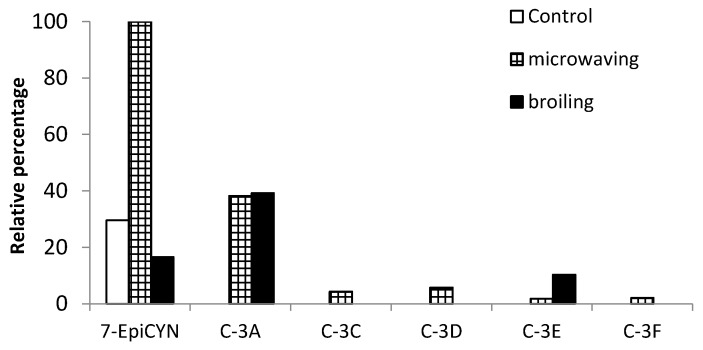
Relative percentage of the CYN decomposition products generated involved in a possible degradation route of CYN in control and cooked samples.

Four deductions can be inferred from the relative abundance of the decomposition products detected (Figure 3). First of all, among the six diasteroisomers observed, five (C-3A, C-3C, C-3D, C-3E and C-3F) are present in samples submitted to microwaving but not in the uncooked ones, suggesting that this cooking procedure could possibly induce CYN degradation through the formation of these *m*/*z* 336.16663 products.

Secondly, two of the six diasteroisomers (C-3A and C-3E) are characteristic of the cooked samples, regardless of the method employed, compared to the uncooked control ones where they were not detected. This is in agreement with the results of Guzmán-Guillén et al. [14] in boiled and steamed fish, who detected two diastereoisomers with *m*/*z* 336.16663 (named C-3A and C-3B) only in the cooked samples. Thirdly, the most predominant diasteroisomer was C-3A (38–39%) regardless of the cooking method used, so this could represent the major degradation route, and only small percentages of the rest were found (2–10%). Finally, the only decomposition product that increases its relative abundance after cooking is 7-epi-CYN, which is present in uncooked samples only in 30% compared to the 100% it reaches in samples cooked by microwaving, whereas after broiling its relative abundance diminishes (17% compared to the 100% after microwaving). This may indicate that one of the major pathways of CYN degradation by microwaving is its epimerization. By contrast, when broiling is used, the formation of *m*/*z* 336.16663 compounds C-3A and C-3E may be the only CYN degradation pathway, compared to microwaving, where the formation of other C-3 diasteroisomers and mainly 7-epi-CYN is also involved.

**Figure 4:**
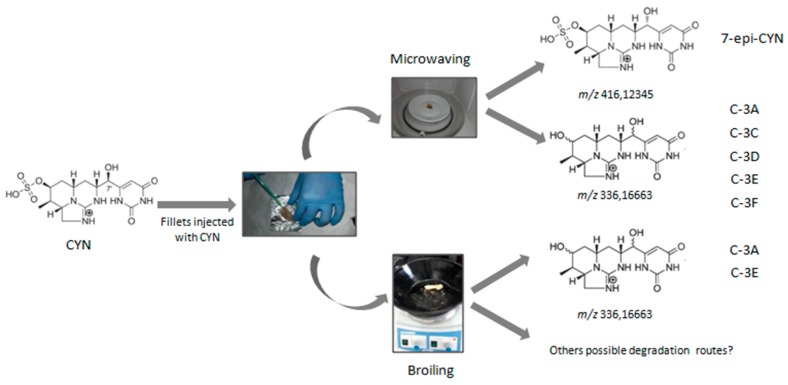
CYN degradation pathways proposals for both cooking processes: microwaving and broiling.

CYN and 7-epi-CYN have six stereocenters, and the difference between the two compounds is in the stereoconfiguration of the hydroxyl group at C-7, with CYN having an R configuration and 7-epi-CYN an S configuration [27]. Moreover, pH, temperature, and other water chemistry parameters may influence epimerization rates and stability [28]. Taking into account that broiling for 2 min is the most effective method evaluated in the present work to reduce CYN content in fish muscle (Figure 1), and the fact that the relative abundance of C-3A is similar in microwaving and broiling (38% and 39%, respectively), we hypothesize that other possible degradation routes not observed in this work might be taking place, which could explain the decrease in CYN content in broiling (Figure 4). Nevertheless, the fact that in the present study the toxin is injected in the ready-to-eat fish fillet represents a first step to further evaluate the risks associated to the consumption of naturally-contaminated fish, and it provides valuable information about how these cooking practices affect CYN content.

## 3. Conclusions

The key outcomes of this study are that: 1) the highest effectiveness in reducing unconjugated CYN levels in cooked fish was obtained by broiling the fish muscle for 2 min (15%), and then by microwaving for 1 (11%) and 2 min (10%); (2) broiling treatment for 1 min did not induce significant reductions in the concentration of CYN in fish; (3) the microwaving and broiling processes generated different decomposition products of CYN with *m*/*z* 336.16663 in fish samples; (4) the possible degradation pathways involved by microwaving may be through the formation of 7-epi-CYN and *m*/*z* 336.16663 compounds, whereas in the case of broiling the last route is the only one involved observed in this study; and finally (5) more studies focused on CYN degradation, and toxicity characterization of these decomposition products would be necessary for a more realistic risk evaluation related to consumption of CYN-contaminated fish. Moreover, in in vivo scenarios, CYN content could be conditioned by different biological and chemical mechanisms, which could show different results after the cooking procedures tested in the present study.

## 4. Materials and Methods

### 4.1. Reagents

Standard of CYN (100 µg/mL, 95% purity) was from Alexis Corporation (Lausen, Switzerland), and was diluted in Milli-Q water as required for working solutions (1–100 µg/L). All chemicals and reagents employed in the present work were of analytical grade. HPLC-grade methanol, dichloromethane, acetonitrile, and trifluoroacetic acid (TFA) were supplied by Merck (Darmstadt, Germany). Deionized water (418 MΩ/cm resistivity) was obtained from a Milli-Q water purification system (Millipore, Bedford, USA). BOND ELUT^®^ carbon cartridges (PGC columns) (500 mg, 6 mL) were purchased from Agilent Technologies (Amstelveen, The Netherlands, Europe) and Bakerbond^®^C18 cartridges (500 mg, 6 mL) from Dicsa (Andalucía, Spain). All reagents used for UHPLC-MS/MS were of LC-MS grade; acetonitrile and water were supplied by VWR International (Fontenay-sous-Bois, France) and formic acid from Fluka (Stainheim, Germany).

### 4.2. Sample Preparation

All animals received humane care in compliance with the guidelines for the protection of animals used for scientific purposes [29], and all procedures were previously accepted by the Ethic Committee of the University of Seville. Nile tilapia (*O. niloticus*) were supplied by Valenciana de Acuicultura (fish hatchery in Valencia, Spain) and kept in the laboratory for a 15-day acclimatization period in two 96-L aquariums (individuals per aquarium: 8) with tap water, at a constant temperature (21 ± 2 °C). For this period, fish were fed daily (0.3 g per day) with commercial fish food (Dibaq S.L., Segovia, Spain). After acclimation, fish were sacrificed, dissected and separated into fillets of 4.00 ± 0.25 g. Five fish fillets per cooking method (*n* = 5) were injected with 500 µL of a CYN stock solution containing 100 µg CYN/L, equivalent to 50 ng CYN/g dry weight (d.w.). This was carefully carried out by repetitive injections in different parts of the fish fillet using a syringe covering the whole area, to ensure a homogeneous intramuscular distribution of CYN in the fish fillet. Samples were left for 20–30 min before being submitted to cooking processes. This concentration of CYN was considered environmentally relevant according to Guzmán-Guillén et al. [14].

### 4.3. Simulation of Food Processing Practices

Four groups with *n* = 5 fillets were formed: three groups were injected with 50 ng CYN/g d.w. (control uncooked group, microwaving group for 1 or 2 min, and broiling group, for 1 or 2 min), and one control group was without CYN and not cooked. The cooking time (1 or 2 min) was selected according to the weight of the fillets (4 g), to avoid unappealing aspects for human consumption. For microwaving, a conventional household microwave oven (Samsung M17-13, 300 W, 2450 MHz) was used. Broiled samples were cooked in Teflon pans for both sides of the fillet, employing half the time per side. Salts, spices or other additional ingredients were not added to the samples. Fillet weights were recorded before (4 ± 0.25 g) and after cooking. For microwaving, the final mean weights of the fillets after cooking were 3.52 ± 0.23 g and 2.25 ± 0.16 g, for 1 and 2 min, respectively. For broiling, mean weights were 3.69 ± 0.10 g and 3.32 ± 0.14 g, for 1 and 2 min, respectively. These results show a mean loss of 28% and 12.5% for microwaving and broiling samples, respectively (without lyophilization). All samples were kept at −80 °C until lyophilization (Cryodos 80, Telstar, Tarrasa, Spain) prior to CYN extraction. The final mean lyophilized weight was 0.88 ± 0.05 g d.w., giving a total weight loss of approximately 78% due to cooking and lyophilization. Values are expressed as ng CYN/g dry weight (d.w.), and represent the unconjugated CYN fraction.

### 4.4. Extraction and Purification of CYN from Fish Fillets

Extraction and clean-up procedures were performed according to the method validated by Guzmán-Guillén et al. [30]. Briefly, 0.5 g of the fish fillets were extracted twice with 20 mL Milli-Q water/acetonitrile (30:70, *v*/*v*) containing 0.5% TFA *v*/*v*. After homogenization with ultraturrax and sonication, the mixture was centrifuged at 20.000 rpm (10 min). The purification step was performed with a double-column system (C-18 and PGC). Finally, extracts were passed through a Nanosep^®^ MG centrifugal device (polypropylene and modified nylon) and a syringe filter (0.45 and 0.22 μm, respectively) and analysed by UPLC-MS/MS.

### 4.5. Chromatographic Conditions

The detection and quantification of CYN by UPLC-MS/MS were done applying the method validated by Guzmán-Guillén et al. [14]. The method employed does not detect protein-bound CYN, so CYN concentrations in the fish fillets represent the un-bound toxin. Separation of the chromatographic peaks was carried out in a UPLC Acquity (Waters, Milford, MA, USA) coupled to a Xevo TQS-micro (Waters) consisting of a triple quadrupole mass spectrometer equipped with an electrospray ion source operated in positive mode. The chromatographic conditions are described in Guzmán-Guillén et al. [14].

### 4.6. Analytical Detection of CYN Decomposition Products after Cooking by UHPLC-MS/MS Orbitrap

For characterization of the possible decomposition products of unconjugated CYN after the influence of cooking, microwaving and broiling procedures were reproduced for 2 min, employing two fillets per cooking method by injecting into them 500 µL of a solution containing 80 µg CYN/mL (equivalent to 40 µg CYN/g d.w.). This was needed because unconjugated CYN contents detected after injecting with 50 ng CYN/g d.w. were too low to be analysed by Orbitrap. This quantity was selected according to that used by Adamski et al. [23] to identify CYN by-products in a *Cylindrosperomposis raciborskii* culture. Afterward, cooking of samples and extraction of CYN were performed as described in Section 4.3 and Section 4.4, respectively. All analyses were performed using a Thermo Scientific liquid chromatography system consisting of a binary UHPLC Dionex Ultimate 3000 RS, connected to a quadrupole-orbitrap Qexactive hybrid mass spectrometer (Thermo Fisher Scientific, Bremen, Germany), equipped with a heated-electrospray ionization probe (HESI-II). Apparatus conditions are detailed in Guzmán-Guillén et al. [14]. Eluent A was water/formic acid (0.1%, *v*/*v*), eluent B was acetonitrile/formic acid (0.1%, *v*/*v*), and injection volume was 5 µL, according to Adamski et al. [22,23].

MS detection of the CYN decomposition products observed by Adamski et al. [25,26] was performed in the present study with the Q-Exactive Orbitrap mass spectrometer through the accurate *m*/*z* measurement of each molecule, by scanning from 100 to 1000 *m*/*z*, in positive and full scan (FS) mode at a resolution of 70,000 (full width half maximum, FWHM at *m*/*z* 200). Each compound observed was associated with a putative chemical formula, including a relative mass error, considered acceptable if <5 ppm [31]. The Q-Exactive Orbitrap was also used to support the identity of CYN decomposition products by tandem mass spectrometry (MS^2^) experiments using the parallel reaction monitoring (PRM) method, which was acquired in positive mode and a resolution of 17,500, with an isolation window of 1 *m*/*z*, and normalized collision energy was set at 35 eV. The selected [M + H]^+^ were 214.15500, 290.08052, 292.09617, 336.16663, 338.18228, 414.10780, 416.12345 and 434.13401. The identity of the compounds was then sustained by the agreement between theoretical and experimental isotopic patterns, and by the product ions formed during MS^2^ analysis. HESI source parameters were: spray voltage 3.5 kV, capillary temperature 320 °C, sheath, auxiliary, and sweep gas flow rate (N_2_) 45, 15 and 2 (arbitrary units) respectively, probe heater temperature 350 °C, and S-Lens RF level 50. A relative comparison between different cooking treatments and the control was performed taking into account the areas of the chromatographic peaks, selecting the following fragments: *m*/*z* 194.12879 for the molecular ions [M + H]^+^ 416.12345 and 336.16663; *m*/*z* 210.12427 for the molecular ion [M + H]^+^ 290.08052; *m*/*z* 212.13935 for the molecular ion [M + H]^+^ 292.09617, and the fragment ion at *m*/*z* 192.11314 for the molecular ion [M + H]^+^ 414.10780.

### 4.7. Statistical Analysis

One-way analysis of variance with Tukey’s post-test was performed verified with the normality test (Kolmogorov–Smirnov) and homogeneity of variances test (Bartlett) using the statistical software INSTAT 3.06, Graph Pad^TM^ (San Diego, CA, USA), representing mean ± standard deviation (SD) of five samples/group (*n* = 5). Differences in mean values between groups were considered statistically significant at *p* < 0.05 level.

## Figures and Tables

**Table 1:** CYN decomposition products in muscle of tilapia fish (*O. niloticus*) cooked by microwaving for 2 min.

Decomposition Products	Retention Time (min)	Putative Chemical Formula	[M + H]^+^ Ion Full Scan (FS)			Fragments Parallel Reaction Monitoring (PRM)			
			*m*/*z* obs.	*m*/*z* calc.	Mass Error (ppm)	Fragment Formula	*m*/*z* obs.	*m*/*z* calc.	Mass Error (ppm)
C-1	1.54	C_10_H_15_N_3_O_5_S	290.08090	290.08052	1.32	C_10_H_15_N_3_O_2_	210.12402	210.12370	1.52
						C_10_H_13_N_3_O	192.11319	192.11314	0.27
C-2A	2.45	C_10_H_17_N_3_O_5_S	292.09595	292.09617	−0.76	C_10_H_17_N_3_O_2_	212.13928	212.13935	−0.34
						C_10_H_15_N_3_O	194.12901	194.12879	1.16
						C_10_H_13_N_3_	ND	176.11822	
C-2B	3.81	C_10_H_17_N_3_O_5_S	292.09634	292.09617	0.60	C_10_H_17_N_3_O_2_	212.13954	212.13935	0.89
						C_10_H_15_N_3_O	194.12900	194.12879	1.08
						C_10_H_13_N_3_	ND	176.11822	
7-epi-CYN	2.87	C_15_H_21_N_5_O_7_S	416.12320	416.12345	−0.59	C_15_H_21_N_5_O_4_	336.16690	336.16663	0.80
						C_15_H_19_N_5_O_3_	318.15652	318.15607	1.44
						C_10_H_15_N_3_O_4_S	274.08606	274.08560	1.67
						C_10_H_15_N_3_O	194.12900	194.12879	1.08
						C_10_H_13_N_3_	176.11835	176.11822	0.70
CYN	3.25	C_15_H_21_N_5_O_7_S	416.12350	416.12345	0.14	C_15_H_21_N_5_O_4_	336.16693	336.16663	0.89
						C_15_H_19_N_5_O_3_	318.15637	318.15607	0.96
						C_10_H_15_N_3_O_4_S	274.08600	274.08560	1.44
						C_10_H_15_N_3_O	194.12901	194.12879	1.16
						C_10_H_13_N_3_	176.11833	176.11822	0.61
C-3A	3.44	C_15_H_21_N_5_O_4_	336.16611	336.16663	−1.56	C_10_H_15_N_3_O	194.12901	194.12879	1.16
						C_10_H_13_N_3_	ND	176.11822	
C-3B	5.06	C_15_H_21_N_5_O_4_	336.16687	336.16663	0.71	C_10_H_15_N_3_O	194.12895	194.12879	0.84
						C_10_H_13_N_3_	ND	176.11822	
C-3C	4.86	C_15_H_21_N_5_O_4_	336.16748	336.16663	2.53	C_10_H_15_N_3_O	194.12903	194.12879	1.23
						C_10_H_13_N_3_	ND	176.11822	
C-3D	4.96	C_15_H_21_N_5_O_4_	336.16660	336.16663	−0.10	C_10_H_15_N_3_O	194.12891	194.12879	0.61
						C_10_H_13_N_3_	ND	176.11822	
C-3E	5.17	C_15_H_21_N_5_O_4_	336.16620	336.16663	−1.28	C_10_H_15_N_3_O	194.12901	194.12879	1.16
						C_10_H_13_N_3_	ND	176.11822	
C-3F	5.27	C_15_H_21_N_5_O_4_	336.16693	336.16663	0.89	C_10_H_15_N_3_O	194.12929	194.12879	2.57
						C_10_H_13_N_3_	ND	176.11822	
C-4	4.94	C_15_H_19_N_5_O_7_S	414.10715	414.10780	−1.56	C_15_H_19_N_5_O_4_	334.15170	334.15098	2.16
						C_10_H_13_N_3_O_4_S	272.07062	272.06995	2.44
						C_10_H_13_N_3_O	192.11334	192.11314	1.06

**Table 2:** CYN decomposition products in muscle of tilapia fish (*O. niloticus*) cooked by broiling for 2 min.

Decomposition Products	Retention Time (min)	Putative Chemical Formula	[M + H]^+^ Ion Full Scan (FS)			Fragments Parallel Reaction Monitoring (PRM)			
			*m*/*z* obs.	*m*/*z* calc.	Mass Error (ppm)	Fragment Formula	*m*/*z* obs.	*m*/*z* calc.	Mass Error (ppm)
C-1	1.52	C_10_H_15_N_3_O_5_S	290.08063	290.08052	0.38	C_10_H_15_N_3_O_2_	210.12390	210.12370	0.94
						C_10_H_13_N_3_O	192.11357	192.11314	2.25
C-2A	2.44	C_10_H_17_N_3_O_5_S	292.09634	292.09617	0.60	C_10_H_17_N_3_O_2_	212.13951	212.13935	0.74
						C_10_H_15_N_3_O	194.12917	194.12879	1.94
						C_10_H_13_N_3_	176.11784	176.11822	2.16
C-2B	3.81	C_10_H_17_N_3_O_5_S	292.09631	292.09617	0.50	C_10_H_17_N_3_O_2_	212.13954	212.13935	0.89
						C_10_H_15_N_3_O	194.12903	194.12879	1.23
						C_10_H_13_N_3_	176.11868	176.11822	2.61
7-epi-CYN	2.88	C_15_H_21_N_5_O_7_S	416.12357	416.12345	0.29	C_15_H_21_N_5_O_4_	336.16693	336.16663	0.89
						C_15_H_19_N_5_O_3_	318.15631	318.15607	0.77
						C_10_H_15_N_3_O_4_S	274.08621	274.08560	2.22
						C_10_H_15_N_3_O	194.12901	194.12879	1.16
						C_10_H_13_N_3_	176.11847	176.11822	1.39
CYN	3.24	C_15_H_21_N_5_O_7_S	416.12369	416.12345	0.58	C_15_H_21_N_5_O_4_	336.16696	336.16663	0.98
						C_15_H_19_N_5_O_3_	318.15643	318.15607	1.15
						C_10_H_15_N_3_O_4_S	274.08603	274.08560	1.55
						C_10_H_15_N_3_O	194.12904	194.12879	1.31
						C_10_H_13_N_3_	176.11835	176.11822	0.70
C-3A	3.44	C_15_H_21_N_5_O_4_	336.16647	336.16663	−0.47	C_10_H_15_N_3_O	194.12904	194.12879	1.31
						C_10_H_13_N_3_	ND	176.11822	
C-3E	5.14	C_15_H_21_N_5_O_4_	336.16721	336.16663	1.71	C_10_H_15_N_3_O	194.12903	194.12879	1.23
						C_10_H_13_N_3_	ND	176.11822	
C-4	5.03	C_15_H_19_N_5_O_7_S	414.10718	414.10780	−1.49	C_15_H_19_N_5_O_4_	334.15115	334.15098	0.52
						C_10_H_13_N_3_O_4_S	272.07028	272.06995	1.21
						C_10_H_13_N_3_O	192.11337	192.11314	1.22
